# Dispensary Abuse

**Published:** 1914-11

**Authors:** 


### Dispensary Abuse.

Michael M. Davis of Boston, Med. Rec., Sept., 12, 1914, considers this a medical bugbear and suggests that the most important element in the conduct of a dispensary is to insure proper treatment of patients. While the statistics of investigation quoted are subject to criticism on the ground that all charity workers are inclined to be too charitable, not to mention the frank avowal that three million persons are treated annually in dispensaries, and that the lowest percentages of unworthy applicants indicate that a tenth should not receive medical charity, we believe that Dr. Davis has shown a weak point in our profession. It is a safe opinion that the patient who can pay a physician and who applies for dispensary treatment would get free or at least, cheap medical treatment if there were no dispensaries. For instance, we have heard of a young millionaire who put on old clothes, left his car a block away from a specialist's office, assumed another name, and got a charity rate. Just so long as we have more physicians than are needed, there will be men willing to work for nothing and willing to violate principles of ethics and decency, not for directly mercenary motives but for the glory of getting clinical material. And, just so long as we have medical men developing with this idea that a patient is "material", the laity, poor and rich alike, will suffer proportionately from poor therapeutics.

There is no getting away from the results of the economic fault of upsetting the law of supply and demand. It makes no difference whether the "abuse" is at the dispensary or at the private office, whether competition exceeds legitimate bounds from need of money or from desire for "material," whether the attempt to get more than the average share of work is made through newspaper advertising or by touts, or by manipulation of institutions, the ultimate cause and the ultimate effect are the same. The profession is doing its best now to attack the root of the evil and, as statistics of medical education show, with success. But the success will be manifest only in the future. For the present, the best way is to do one's own best and not to worry about competition.

